# Contributions of a Histone Deacetylase (SirT2/Hst2) to *Beauveria bassiana* Growth, Development, and Virulence

**DOI:** 10.3390/jof8030236

**Published:** 2022-02-27

**Authors:** Qing Cai, Li Tian, Jia-Tao Xie, Dao-Hong Jiang, Nemat O. Keyhani

**Affiliations:** 1 College of Plant Science and Technology, Huazhong Agricultural University, Wuhan 430070, China; jiataoxie@mail.hzau.edu.cn (J.-T.X.); daohongjiang@mail.hzau.edu.cn (D.-H.J.); 2 Department of Microbiology and Cell Science, University of Florida, Bldg. 981, Museum Rd., Gainesville, FL 32611, USA; 3 Shandong Provincial Key Laboratory of Microbial Engineering, Department of Bioengineering, Qilu University of Technology, Jinan 250353, China; tianliql@163.com

**Keywords:** histone deacetylation, sirtuin, virulence, conidiation, cell cycle, metabolism, LysM effectors, polyketide synthases

## Abstract

Sirtuins are a class of histone deacetylases that promote heterochromatin formation to repress transcription. The entomopathogenic fungus *Beauveria bassiana* contains six sirtuin homologs. The class III histone deacetylase, BbSir2, has been previously shown to affect the regulation of carbon/nitrogen metabolism and asexual development, with only moderate effects on virulence. Here, we examine another class III histone deacetylase (BbSirT2) and show that it contributes to deacetylation of lysine residues on histone H4-K16ac. Directed gene-knockout of *BbSirT2* dramatically reduced conidiation, the ability of the fungus to metabolize a range of carbon and nitrogen sources, and tolerances to oxidative, heat, and UV stress and significantly attenuated virulence in both intrahemocoel injection and topical bioassays using the Greater wax moth (*Galleria mellonella*) as the insect host. Δ*BbSirT2* cells showed alterations in cell cycle development and hyphal septation and produced morphologically aberrant conidia. Comparative transcriptomic analyses of wild type versus Δ*BbSirT2* cells indicated differential expression of 1148 genes. Differentially expressed genes were enriched in pathways involved in cell cycle and rescue, carbon/nitrogen metabolism, and pathogenesis. These included changes in the expression of polyketide synthases (PKSs) and LysM effector proteins that contribute to degradation of host toxins and target host pathways, respectively. These data indicate contributions of BbSirT2 in helping to mediate fungal stress and development, with the identification of affected gene targets that can help account for the observed reduced virulence phenotype.

## 1. Introduction

Histone acetylation and deacetylation are post-translational protein modifications that impact core molecular processes of gene expression by mediating transitions between transcriptionally repressed and active chromatin states [1,2,3]. Histone deacetylation is mediated by histone deacetylases (HDACs), a multifamily enzyme family found in all eukaryotes, capable of catalyzing the deacetylation of lysine residues (Kac sites) on histones and other proteins [4,5,6]. Different subfamilies of histone deacetylases (HDACs) have been characterized, including class I and class II enzyme families, e.g., the Rpd3/Hda1-like HDACs and the class III NAD^+^-dependent sirtuins [5,7]. Amino acid lysine (K) acetylation catalyzed by HDACs can also occur on non-histone targets, directly affecting cell processes ranging from transcription to signaling and response pathways and protein synthesis, affecting cell cycle progression, metabolism, stress and immune responses, and, in the case of pathogens, virulence [3,8].

In addition to catalyzing the protein deacetylation reaction, sirtuins can mediate the removal of succinyl, malonyl, myristoyl, and/or palmitoyl groups from amino acid side chains [9,10,11]. Sirtuins also include a subfamily of mono-ADP-ribosyltransferases. In the yeast *Saccharomyces cerevisiae,* Sir2, originally identified as the silent mating type information regulator 2 protein, along with four other Sir2 paralogues, Hst1–Hst4, represent the NAD-dependent histone deacetylase repertoire found in this organism [12,13,14,15,16,17,18]. *S. cerevisiae* Hst2 is a highly conserved member of the Sir2 family, encoding for an NAD^+^-dependent deacetylase with its closest human homolog being SIRT2 [19,20,21]. Yeast Homolog of SIR Two 2 (yHst2) activity represents the majority of NAD^+^-dependent deacetylase activity detected in yeast cell extracts [22], with yHst2 exhibiting higher in vitro activity using histone substrates than ySir2 [13]. Structurally, yHst2 consists of a conserved catalytic domain and less-conserved carboxy and amino-terminal domains that inhibit NAD^+^ and acetyl–lysine binding, respectively [23]. yHst2 is mainly localized to the cytoplasm but contains a leucine-rich nuclear export sequence that permits the protein to traffic between the nucleus and cytoplasm [19,24]. In mice, SirT2 (a yHst2 ortholog) was found to be mainly localized to the nucleus during the early prophase and mitosis and was shown to primarily act to deacetylate H4K16Ac in vitro [25].

yHst2 has been shown to deacetylate histones in vivo through direct binding to promoter regions, e.g., yHst2 has been shown to participate in the repression of the *flocculation 10* gene (*FLO10*) involved in regulation of stress and flocculation [20,25]. Furthermore, overexpression of yHst2 affects recombination and transcriptional silencing, and yHst2 can directly regulate the expression of rDNA genes under conditions of calorie restriction, thus playing a role in extending the replicative life span (RLS) [19,26]. yHst2 levels are influenced (positively) during stationary phase growth via its association with a number of stress-related proteins as well as the Hos2 nuclear histone deacetylase [27]. Moreover, nuclear yHst2 has been shown to interact with chromatin to downregulate the expression of sub-telomeric genes along with Hst1 [19,20].

In fission yeast, Hst2 is predominantly cytoplasmic; however, a nuclear pool of Hst2 has been shown to colocalize with other sirtuins in heterochromatic regions, e.g., centromeres, telomeres, and mating-type loci, and also can affect the silencing of rDNA genes [28]. In the human pathogenic fungus *Candida albicans*, Hst2 is mainly localized to the cytoplasm and contributes to fungal growth as well as to the dimorphic transition that occurs during growth within hosts, in collaboration with Sir2 and Hst1 [29]. Similarly, in the human pathogen *Cryptococcus neoformans*, SirT2 contributes to cell cycle control, lifespan, and virulence [30]. In the saprophytic fungus *Podospora anserina*, under dietary restriction (DR) conditions, deletion of the *Sir2* or *Hst2* genes induced a significant reduction in life span extension, while loss of both *Sir2* and *Hst2* strongly reduced life span extension [31].

The entomogenous fungus *Beauveria* *bassiana* is a widely used commercialized fungal biological pest control agent used for the control of insects affecting agricultural crops worldwide, with applications towards the control of invasive forestry pests and human/livestock disease-causing agent vectoring insects, i.e., ticks and mosquitoes [32,33]. In part, the efficacy of *B. bassiana* depends on the conidial spore production and the viability and tolerances of the conidia to abiotic and biotic stresses (temperature, humidity, pH). In addition, the production of a variety of virulence factors can affect host range and insect mortality rates [34,35]. Processes impacting development and virulence are regulated by genes involved in hyphal growth, asexual development, signal transduction in response to host cues, cell cycle control, and carbon/nitrogen utilization, along with factors that affect multistress tolerances and detoxification of host and environmental toxins [36]. Thus far, a single class III histone deacetylase, *Beauveria bassiana* Sir2 (BbSir2), has been previously shown to affect the regulation of carbon and nitrogen metabolism and asexual development, with overall only moderate effects on virulence [37]. Mutation of the *BbSir2* gene resulted in an array of pleiotropic effects, including on virulence, where, in topical insect bioassays, virulence was decreased by ~30%, whereas virulence was decreased by only ~25% in intrahemocoel injection assays [37]. The roles of the remaining sirtuins in *B. bassiana* are unknown. SirT2 homologs have been shown to have distinct roles in cargo-loading, the release of extracellular vesicles, and the control of neurodegenerative diseases, and have even been implicated as a novel therapeutic target for age-related disorders [38,39,40]. We therefore sought to characterize the SirT2/Hst2 homolog in *B. bassiana*. Our data show that *B. bassiana* SirT2 (BbSirT2) has more pronounced (as compared with BbSir2) effects on distinct cellular targets that impact conidiation, metabolism, stress tolerances, cell cycle progression, and virulence. With respect to the latter phenotype (virulence), in contrast to the effects seen for the *BbSir2* mutant, the Δ*BbSirT2* strain was significantly less virulent when assayed using either intrahemocoel injection or topical spray bioassays, and, unlike the former (Δ*BbSir2*) mutant, the Δ*BbSirT2* mutant was almost completely unable to sporulate on host cadavers. These data indicate significant divergence in sirtuin functioning. Transcriptome studies revealed global changes in gene expression resulting from loss of *BbSirT2*, particularly in the expression of genes involved in metabolism, the cell cycle, and virulence. These included genes involved in growth on various substrates, genes that help mediate stress tolerances, and genes that act during the remediation of insect antimicrobial defensive compounds and toxins, as well as effector proteins implicated in virulence.

## 2. Materials and Methods

### 2.1. Bioinformatic Analysis of BbSirT2

The amino acid sequences of *S. cerevisiae* and *A. nidulans* SirT2/Hst2 sirtuins (ScHst2: NP_015310; AnSirT2: XP_680730) were used to search for homologs in the *B. bassiana ARSEF 2860* genomic database (NCBI accession: NZ_ADAH00000000) [41] as well as other representative fungi, including fungal plant, insect, and human pathogens (http://blast.ncbi.nlm.nih.gov/, accessed on 16 January 2014). Amino acid sequences of the set of sirtuin HDAC homologs were aligned with the SMART program (http://smart.embl-heidelberg.de, accessed on 16 January 2014) for structural comparison and by the MEGA7 software (http://www.megasoftware.net, accessed on 16 January 2014) for phylogenetic analysis. The isoelectric point and molecular weight of BbSirT2 were predicted using the ExPASy-Compute pI/Mw online tool (https://web.expasy.org/compute_pi/, accessed on 16 January 2014).

### 2.2. BbSirT2 Subcellular Localization

The open reading frame (ORF) of *BbSirT2* was PCR-amplified from cDNA derived from wild-type *B. bassiana* using the cSirT2-F/R primer pair (Table S1), and the *GFP* protein (GenBank accession code: U55763) was fused to the C-terminus of BbSirT2 as described in [37]. The recombinant gene *BbSirT2::GFP* was cloned into the plasmid pAN52-*bar* (phosphinothricin resistance) and transformed into the *B. bassiana* wild type [42]. Putative transformants expressing the fusion protein product were plated on phosphinothricin (200 μg mL^–1^). After single-colony purification, transformants were examined by confocal microscopy (LSCM, FLUOVIEW FV3000, OLYMPUS). Fungal conidia were grown in SDB (4% glucose, 1% peptone, and 1% yeast extract) at 25 °C for 3 days with aeration at 150 rpm. Growing (hyphal) cells were harvested and counter-stained with Hoechst 33258 dye (DNA-specific, Sigma, St. Louis, MO, USA) for 30 min at room temperature for microscopic visualization.

### 2.3. Construction of BbSirT2 Deleted and Complemented Strains

The deletion strain of *BbSirT2* was constructed using 5′ and 3′ coding fragments (1579 and 1576 bp, respectively) flanking the *bar* selection marker for and subsequent generation of a targeted gene deletion strain via transformation and homologous recombination. The complemented strain (Δ*BbSirT2::BbSirT2*) was constructed by transformaton and ectopic integration of the full-length *BbSirT2* sequence with ~1500 bp of the 5′-flanking (promoter) region (5102 bp total) cloned into a *sur* sulfonyl urea resistance marker containing vector as described in [37,43]. DNA fragments were PCR-amplified from genomic *B. bassiana* DNA using the primers listed in Table S1 with La*Taq* DNA polymerase. Δ*BbSirT2* mutant colonies were initially selected on phosphinothricin (200 μg mL^–1^) plates, or on chlorimuron ethyl (10 μg mL^–1^) plates for the Δ*BbSirT2::BbSirT2* strain. Validation of the correct mutant in complementation integration events in transformed *B. bassiana* strains was performed by PCR and Southern blotting (Table S1).

### 2.4. Histone Acetylation Detection

Fungal strains (wild type, Δ*BbSirT2*, and Δ*BbSirT2::BbSirT2*) were grown in SDB media for 3 d (25 °C, aeration), after which the total protein content was extracted from the cultures for measurement of H4-K16ac protein acetylation by Western blotting using protocols essentially as described previously [37]. Protein (total) concentrations were quantified using the BCA Protein Assay Kit (KeyGen, Nanjing, China). As internal controls, levels of β-actin and H4 were determined in blots probed with 1000-fold dilutions of anti-β-actin (Cell Signal Technology, Shanghai, China) or anti-histone H4 (Merck Millipore, Shanghai, China) antibodies. H4-K16ac acetylation was detected using 1000-fold dilution of an anti-acetyl-H4-K16ac antibody (Merck Millipore). Blots were incubated with the primary antibody, washed, and then probed with 5000-fold dilutions of a goat anti-rabbit IgG antibody (Boster, Wuhan, China). Reactive bands were detected by chemiluminescence visualization (Amersham Biosciences, Shanghai, China). Experiments were repeated three times. Western blot signal intensities were quantified using the ImageJ software (https://imagej.nih.gov/ij/, accessed on 23 June 2014) and the data used to determine the ratios of histone acetylation on each site.

### 2.5. Phenotypic Experiments

To assess effects on fungal growth, aliquots (1 μL) of conidial suspensions (1 × 10^6^ conidia mL^–1^) were spotted on agar media plates. Media tested included CZA (3% sucrose, 0.3% NaNO_3_, 0.1% K_2_HPO_4_, 0.05% KCl, 0.05% MgSO_4_, and 0.001% FeSO_4_ plus 1.5% agar), 14 different CZA-derived media prepared by replacing sucrose in the media with either acetate (NaAc), glycerol, fructose, glucose, lactose, trehalose, maltose, or glycerol or by replacing the nitrogen source (NaNO_3_) with either NaNO_2,_ NH_4_Cl, NH_4_NO_3,_ β-alanine, or N-acetylglucosamine (GlcNAc). Plates were incubated at 25 °C for 7 d after inoculation, and colony diameters were subsequently measured. Responses to different stress-causing agents were assessed by spotting conidial suspensions (1 μL, 1 × 10^6^ conidia mL^–1^) onto CZA plates supplemented with either (i) CZA unamended (control), (ii) H_2_O_2_ (2 mM) or menadione (0.02 mM) (oxidative stress), (iii) 0.4 M NaCl, 0.4 M KCl, or 0.8 M sorbitol (osmotic stress), (iv) H_2_O_2_ (2 mM) or menadione (0.02 mM) (oxidative stress), (v) carbendazim (CBD) (10 μg mL^–1^) or iprodione metabolite (IPM, 10 μg mL^–1^) (drug resistance analysis), (vi) hydroxyurea (HU) (10 mM) or methyl methanesulfonate (MMS) (0.05%) (DNA damage stress), or (vii) SDS (100 μg mL^–1^) or Congo Red (10 μg mL^–1^) (cell-wall-perturbing stress). Colony diameters were measured 7 d post-inoculation after growth at 25 °C. Effects on inhibiton of growth by each respective chemical agent tested were calculated using the equation (*S*_c_–*S*_t_)/*S*_c_ × 100, where *S*_c_ and *S*_t_ denote growth under control and test conditions, respectively.

To examine effects on conidial production, aliquots (100 μL, 1 × 10^7^ conidia mL^–1^) were spread evenly on SDAY plates (9-cm diameter Petri plates) and incubated at 25 °C for 9 d using a light/dark cycle of 12:12 h. Starting at Day 4 of growth, three 5-mm diameter agar plugs were removed from each plate/day, and the conidia/plug were washed into 1 mL of 0.02% Tween 80 by ultrasonication. The resultant conidia were counted using a hemocytometer and the number of conidia was converted to conidia/cm^2^. Conidia and conidial structures were visualized by scanning electron microscopy (SEM) from cultures after 5 and 7 d of growth on SDAY plates as described previously [37]. Conidial UV-B resistances, thermotolerances, and viability were assessed and calculated as: (i) the time required to observe 50% conidial germination (GT_50_, h) at 25 °C (viability); (ii) the mean lethal time to kill 50% of the conidia (LT_50_, min) in response to a wet-heat stress at 45 °C (thermotolerance); and (iii) the mean lethal dose to kill 50% of the conidia (LD_50_, J cm^–2^) (response to UV-B irradiation), essentially as previously described [37].

Insect bioassays were performed using *G. mellonella* larvae with two different methods of infection: (i) intrahemocoel injection assays in which 5 μL of a 1 × 10^5^ conidia mL^–1^ suspension was injected into the haemocoel of each larvae; and (ii) topical (cuticular) bioassays in which larvae were immersed for 10 s in a 30 mL suspension of 1 × 10^7^ conidia mL^–1^. Insects (30/replicate, 3 replicates/experiment) were maintained at 25 °C and examined every 12 or 24 h for mortality over a 10-d time course. The entire experiment was repeated three times. The mean lethal time to kill 50% of the hosts (LT_50_) was calculated using Probit analyses. Fungal hyphal bodies produced within the insect hemocoel were examined microscopically from hemolymph aliquots taken from larvae 96 and 120 h post-treatment. Images of insect cadavers (4 d post-death) were also taken to examine fungal (out)growth from infection.

Activities of proteases and other extracellular enzymes implicated in insect cuticle degradation and/or fungal virulence [44,45] were quantified for each strain as previously detailed [46]. Briefly, fungal cultures (100 mL) were grown in CZB + 0.3% bovine serum albumin and incubated at 25 °C for 4 d with aeration. Fungal biomass was harvested by centrifugation (13,200× *g* for 1 min at 4 °C) and quantified (mg mL^–1^). Protease activity was determined using 100 μL of azocasein (Sigma) solution (5 mg mL^−^^1^) mixed with 100 μL of crude extract and incubated for 1 h at 37 °C. Reactions were terminated by adding 400 μL of 10% (*w*/*v*) trichloroacetic acid, and samples were centrifuged at 12,000× *g* for 5 min, with the supernatant transferred to tubes containing 700 μL of 525 mM NaOH. Samples were read at an optical density (OD) = 442 nm using a spectrophotometer. Pr1 protease activity was determined in reaction mixtures containing 50 μL of 1 mM succinyl-(alanine)_2_-proline-phenylalanine-p-nitroanilide (Sigma), 850 μL of 15 mM Tris HCl buffer (pH 8.5), and 100 μL of the crude extract. The reaction mixture was incubated for 1 h at 28 °C and terminated by addition of 250 μL of 30% acetic acid. Samples were kept on ice for 15 min then centrifuged at 12,500× *g* for 5 min at 4 °C, and the resultant supernatant was read at OD_410_. One unit of enzyme activity was defined as the amount of enzyme needed to increase the OD by 0.01/1 h of reaction time, with total activity expressed as U mg^–1^ biomass.

### 2.6. Investigation of Hyphal Septation, Cell Size, and Cell Cycle Progression

Fungal hyphae were isolated from SDB cultures (100 mL) inoculated with 50 mL of a 1 × 10^6^ conidia mL^–1^ suspension and grown at 25 °C for 3 d with aeration. Hyphal cells were stained with calcofluor white (Sigma) for 15 min and examined by fluorescence microscopy. Cell width and length were measured from ~50 hyphal cells/strain using the ImageJ software. Unicellular blastospores were isolated for cell cycle analysis after growth (50 mL cultures, inoculated at 1 × 10^6^ conidia mL^–1^) in NLB media consisting of 4% glucose, 0.4% NH_4_NO_3_, 0.3% KH_2_PO_4_, and 0.3% MgSO_4_. Cutlures were incubated at 25 °C for 3 d with aeration and blastospores were collected via filtration through cheese cloth. The resultant cells were used for determination of cell cycle G_0_/G_1_, G_2_/M, and S phase time lengths using the respective readings of unduplicated (1C), duplicated (2C), and intermediate DNA concentrations via flow cytometry, with three samples per strain examined as previously described [37]. Blastospore density and size were also measured using the FSc and SSc readings derived from the flow cytometry analyses, respectively.

### 2.7. Quantification of Intracellular Mitochondrial and ATP Content

Blastospores were harvested as described above. Samples (1 mL) containing 1 × 10^7^ blastospores/mL (as determined by counting in a hemocytometer) were harvested by centrifugation (10,000× *g*, 5 min) and resuspended in 200 μL of lysis buffer (luciferase-based kit, Beyotime, Nantong, China) as previously described [47]. The resultant suspension was subjected to centrifugation at 12,000× *g* for 5 min at 4 °C, and the supernatant was transferred to a new tube on ice. Aliquots (20 μL) of the blastospore-derived supernatant were mixed with 100 μL of working buffer from the kit. ATP content (total) was quantified using a luminometer (Varioskan Flash, Thermo Scientific, Shanghai, China), and calculated as nM ATP/mg extract. Three techincal replicates were examined for each strain.

Mitochondrial content in blastospores was assessed by suspending the cells in 0.02% Tween 80 containing 500 nM Mito-Tracker Green (a mitochondrial membrane stain, Molecular Probes, Invitrogen, Karlsruhe, Germany). Cells were incubated at room temperature for 15 min, and blastospores were collected by centrifugation (10,000× *g*, 5 min), washed once, and then resuspended in 10 mM PBS buffer (pH 8.0). Fluorescence intensity was quantified via flow cytometry using at least 2 × 10^4^ cells per sample (excitation/emission wavelengths = 490/516 nm). Hyphal cells were also examined via staining (15 min, room temperature) with a solution of 500 nM Mito-Tracker Red (Molecular Probes, Invitrogen, Karlsruhe, Germany). After staining, cells were washed three times using PBS and then resuspended in PBS. Mitochondrial morphology and distribution were observed via confocal fluorescence microscopy (LSM 700, ZEISS). For data analyses, all phenotypic data were subjected to one-way ANOVA followed by Tukey’s honest significant difference (HSD) test.

### 2.8. Gene Expression Analyses of BbSirT2 and Development/Septation Genes

For each test strain, SDAY plates were inoculated with 100 μL of a 1 × 10^7^ conidia mL^–1^ suspension, and the plates were incubated over a time course (2–7 d) at 25 °C. Fungal cells were harvested via scraping of the total cell biomass into eppendorf tubes. Total RNA was isolated using the RNAiso Plus Kit (TaKaRa, Dalian, China) and cDNA was synthesized via reverse transcription using the PrimeScriptH^RT^ reagent kit (TaKaRa). Transcript levels of select genes were examined in the cDNA samples by qPCR with primers designed for each gene (Tables S1 and S2). qPCR reactions were performed using SYBR^®^ Premix Ex Taq^TM^ (TaKaRa). Quantification of the *B. bassiana* 18S rRNA was used as the internal standard. The 2^−ΔΔCt^ method [48] was used to calculate relative transcript levels for each target gene. All experiments included three technical replicates and the entire experiment was performed three times.

### 2.9. Global Transcriptomic Analysis

Fungal cultures (Δ*BbSirT2* and wild type, three replicates for each) were grown on SDAY plates for 4 d, after which the fungal biomass was harvested as describd above. Samples were sent to Huada Gene Company (Shenzhen, China) for the construction and analysis of transcriptome libraries. Briefly, total RNA was extracted from each culture separately (six samples total), and the mRNA pool was extracted using magnetic oligo(dT) beads. After fragmentation, first-strand cDNA was synthesized using random hexamer primers and the mRNA as the template. Second-strand cDNA was then synthesized using the first-strand cDNA as the template. Final cDNAs were purified and then repaired with single adenines added to the ends, after which adaptors were added to the cDNA ends, and the resultant products were sequenced using the Illumina HiSeq 2500 platform. Raw reads were filtered to generate clean tags, and sequences were mapped to the *B. bassiana* genome [41] using significance levels of Log_2_(Δ*SirT2*/WT ratio) <−2 (downregulated) or >2 (upregulated) and a false discovery rate (FDR) < 0.001. Data were normalized to fragments/kilobase of exon/million fragments mapped (FPKM). Differentially expressed genes (DEGs) were functionally annotated with known or putative genes using the non-redundant NCBI protein databases and subjected to FunCat categories classification (https://elbe.hki-jena.de/fungifun/, accessed on 16 July 2019) and Kyoto Encyclopedia of Genes and Genomes (KEGG) analysis (http://www.genome.jp/kegg/, accessed on 16 July 2019) at a significance level of *p* < 0.05. The transcriptome data have been deposited in the NCBI Sequence Read Archive (SRA) (https://submit.ncbi.nlm.nih.gov/subs/sra/, accessed on 22 December 2020) with the dataset identifier PRJNA687276.

## 3. Results

### 3.1. Bioinformatic Analysis of BbSirT2 and Construction of Deleted and Complemented Strains

The BbSirT2/Hst2 homolog in *B. bassiana* was identified as being encoded by a 2589-bp nucleotide sequence that contained four introns (NCBI accession code: EJP65279; tag locus: BBA_05610). Translation of the deduced open reading frame (ORF) resulted in a protein consisting of 520 amino acids (molecular mass: 57.2 kDa; isoelectric point: 4.93) and containing a SIRT1_Super family domain (SIRT1_SF, residues 153–388) typical for the SirT2/Hst2 super family. The *B. bassiana* protein showed a high degree of similarity to the SirT2/Hst2 homologs found in *S. cerevisiae* (48%) and *Aspergillus nidulans* (62%). However, BbSirT2/Hst2 harbors an H4 super family domain (H4_SF, residues 9–151), which is absent in both the *A. nidulans* and *S. cerevisiae* proteins (Figure S1A). Overall, BbSirT2 shared 41%–85% sequence identity with homologs found in filamentous fungi and yeasts (Figure S1B). For functional characterization, a targeted gene deletion mutant (Δ*BbSirT2*) and a subsequent complemented (Δ*BbSirT2::SirT2*) strain were constructed as detailed in the Methods section. The integrity of the *BbSirT2* mutant and complemented strains was verified via PCR and Southern blotting analyses (Figure S2; the primers used are shown in Table S1).

### 3.2. BbSirT2 Nuclear Localization, Histone Deacetylation Target, and Contributions to Conidiation

**Figure 1:**
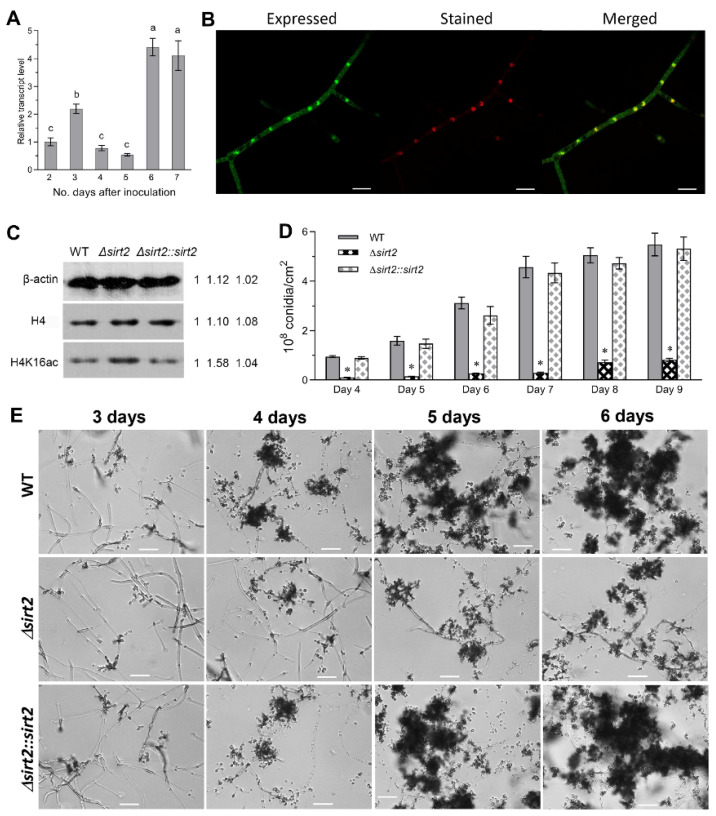
Expression and localization of BbSirT2 and the impact of *BbSirT2* deletion on histone H4-K16 acetylation and conidiation capacity in *B. bassiana*. (**A**) qPCR-determined gene expression analyses of *BbSirT2* in wild-type cultures over the indicated time course during growth on SDAY at 25 °C (normalized to the expression seen on Day 2). (**B**) Fluorescent LSCM images of the GFP-tagged BbSirT2 fusion protein expressed in the wild-type strain. Cells (hyphae) were collected from SDB cultures (day 3) and counter-stained with Hoechst 33258 nuclear dye (scale bars = 10 µm). (**C**) Western blots of extracts from wild-type, *BbSirT2* deleted, and *BbSirT2* complemented strains probed with the indicated antibodies. (**D**,**E**) Quantification of conidial production and images of conidiation derived from growth on SDAY plates as detailed in the Methods section (scale bars = 20 µm). Letters and asterisks indicate a significant difference from other samples (Tukey’s HSD test; * indicates *p* < 0.05). Error bars = ±SD from three replicates.

The expression of the *BbSirT2* gene was examined in the wild-type strain over a time course that included initial mycelia growth (1–2 d post-inoculation), onset of initiation of conidiation (3–4 d), and subsequent conidial maturation (5–7 d). *BbSirT2* expression increased (~2 fold from day 2) in SDAY media after 3 d of growth and increased (~3–5 fold) after 6–7 d of cultivation (Figure 1A).

BbSirT2 subcellular localization was examined using a transgenic strain expressing a BbSirT2::GFP fusion protein. Laser scanning confocal microscopy (LSCM) of fungal cells (Sabouraud dextrose broth, SBD, 3 d) co-stained with the DNA-specific Hoechst 33258 dye (red fluorescence) revealed (green) fluorescence in both the nuclei and cytoplasm of cells, although some enrichment in the nuclear faction was noted (Figure 1B).

In order to examine the effects of BbSirT2 in mediating deacetylation of histone H4-K16 sites, crude extracts were prepared from the wild type, Δ*BbSirT2*, and Δ*BbSirT2::BbSirT2* strains as detailed in the Methods section. Western blots of cell extracts were probed using β-actin, anti-histone H4, and H4-K16 antibodies (Cell Signaling Technology or Merck Millipore). Subsequent densitometric quantification of the Western blots indicated hyperacetylation (i.e., an approximate ~50% increase) of H4-K16ac sites in the Δ*BbSirT2* mutant compared with the wild-type and complemented strains (Figure 1C).

**Figure 2:**
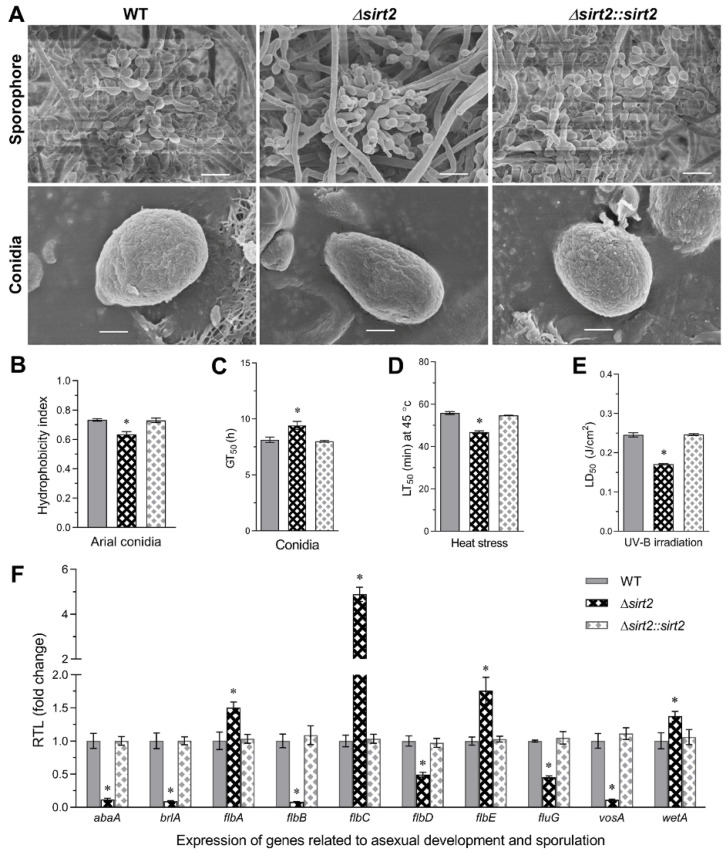
Effect of *BbSirT2* deletion on conidial properties and the expression of asexual-development-related gene pathways. (**A**) SEM images of sporophore structures and conidia (5- and 7-d SDAY plates, respectively). Scale bars: upper panels, 5 µm; bottom panels, 0.5 µm. (**B**) Hydrophobicity of conidia (H-index) quantified as detailed in the Methods section. (**C**–**E**) Conidial germination time (GT_50_), tolerance to heat stress (lethal time, LT_50_), and tolerance to UV-B irradiation (lethal dose, LD_50_), respectively. (**F**) Gene expression levels of ten conidiation-related genes (SDAY media, 3-d cultures) normalizing the expression of the *BbSirT2* mutant strain to the wild type. Asterisks indicate a significant difference from unmarked bars (Tukey’s HSD test; * indicates *p* < 0.05). Error bars = ±SD from three replicates.

The Δ*BbSirT2* strain was severely (*p <* 0.001) impaired in terms of conidial production (an ~90% decrease) over the entire time course examined (3–9 d on SDAY media, Figure 1D,E). Consistent with the overall conidial production data, microscopic visualization of growing mycelia (SDAY media, 5 d) revealed more extensive hyphae but fewer sporophores in the Δ*BbSirT2* mutant as compared with the wild-type strain, with the sporophores observed to apparently be not fully differentiated and appearing distorted and oblong in shape in the Δ*BbSirT2* strain compared with the WT (Figure 2A). Conidia produced by the Δ*BbSirT2* mutant showed lowered hydrophobicity, a critical parameter mediating the attachment of spores to surfaces (Figure 2B). Δ*BbSirT2* conidia showed a small increase in germination time (~15%, Figure 2C, *p <* 0.001). Δ*BbSirT2* conidia were also moderately (*p <* 0.001) more sensitive to UV irradiation and heat stress (~15%–30%, Figure 2D,E). The expression of a suite of ten genes, including the *BlrA* → *AbaA* → *WetA* central conidial pathway components, the *FlbA* regulator of G-protein signaling, the *FlbB–E* genes that include conidiation transcription factors and developmental regulators, *FluG*, which codes for an enzyme considered to produce an extracellular conidiation-inducing signal, and the *VosA* conidial maturation factor, was examined in order to determine whether the lowered conidial yield was due to mis-regulation of conidiation-related genes [49,50]. These data indicate significant repression of *BrlA*, *AbaA,* and *VosA* (*p <* 0.001) expression, whereas the expression of *WetA* was slightly upregulated in the mutant as compared with the wild-type and complemented strains (Figure 2F). Furthermore, expression of the *FlbB*, *FlbD*, and *FluG* genes was found to be downregulated, while *FlbC* and *FlbE* expression levels were significantly higher in the Δ*BbSirT2* mutant as compared with the wild type.

### 3.3. Role of SirT2 in Carbon/Nitrogen Metabolism and Stress Responses

**Figure 3:**
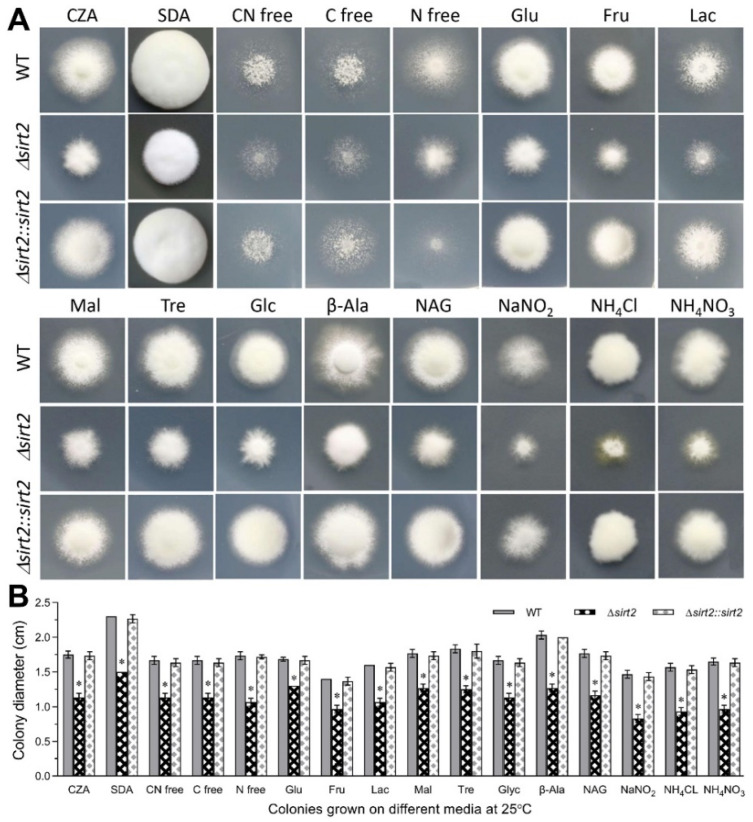
Contribution of BbSirT2 to the utilization of various carbon and nitrogen sources. (**A**) Representative images of fungal colonies and (**B**) measured fungal colony diameters after 7 d of growth at 25 °C on the media detailed in the Methods section. Carbon sources examined: Mal, maltose; Tre, trehalose; Glc, glycerol; Glu, glucose; Fru, fructose; and Lac, lactose. Nitrogen sources examined: NaNO_2_, NH_4_Cl, NH_4_NO_3_, β-Alanine (β-Ala), and N-Acetylglucosamine (NAG). Additional media tested: carbon and nitrogen-free (CN free) media, carbon-free (C free) media, and nitrogen-free (N free) media. All plates were inoculated by spotting (1 μL) aliquots of a 1 × 10^6^ conidia/mL suspension in the center of the plate. All experiments were performed with three technical triplicates. Error bars = ±SD. Asterisks indicate a significant difference from unmarked bars (Tukey’s HSD test; * indicates *p* < 0.05).

To examine the role of BbSirT2 in mediating aspects of fungal development and growth, the wild-type, Δ*BbSirT2*, and complemented strains were grown in different media, including SDAY media, Czapek Dox agar (CZA), and CZA modified with different nitrogen and carbon sources as detailed in the Methods section (Figure 3A,B). Severe growth defects were seen for most of the tested conditions. Growth of the Δ*BbSirT2* mutant was reduced by ~40%–60% on SDAY media, CZA, and CZA amended by six different carbohydrate/polyol carbon sources (fructose, glucose, glycerol, lactose, maltose, and trehalose) as compared with that seen for the wild-type strain (7 d of growth at 25 °C). Similarly, growth on media amended by different nitrogen sources, including three inorganic nitrogen sources (NaNO_2_, NH_4_CL, and NH_4_NO_3_) and two organic nitrogen sources (β-Alanine and N-Acetylglucosamine), was reduced by ~56%–68% when comparing the Δ*BbSirT2* mutant to the control strains (Figure 3A,B).

In order to probe effects on fungal stress tolerances, chemical agents resulting in either oxidative damage, osmotic damage, DNA damage, or cell wall perturbation were tested in agar media. Ablation of *BbSirT2* resulted in higher sensitivity to three osmotic-stress-causing agents (NaCl, sorbitol, and KCl (23%–26%, *p <* 0.001)) and two oxidative-stress-causing agents (menadione (MND) and H_2_O_2_ (27%–34%, *p <* 0.001)) as well as to the two DNA-damage-causing agents tested, namely methyl methanesulfonate (MMS) and hydroxyurea (HU) (18%–30%, *p <* 0.001) (Figure S3A,B). Moreover, the Δ*BbSirT2* mutant also showed a slightly lower but significant reduction in conidial tolerances to the two cell-wall-perturbing agents tested, namely Congo Red and SDS (15%–17%, *p <* 0.001). In addition, a significant (17%, *p <* 0.001) reduction in tolerance to the fungicide iprodione metabolite (IPM) was seen; however, no significant effects were seen in media containing the fungicide carbendazim (CBD) (Figure S3A,B).

### 3.4. Contribution of BbSirT2 to B. bassiana Virulence

**Figure 4:**
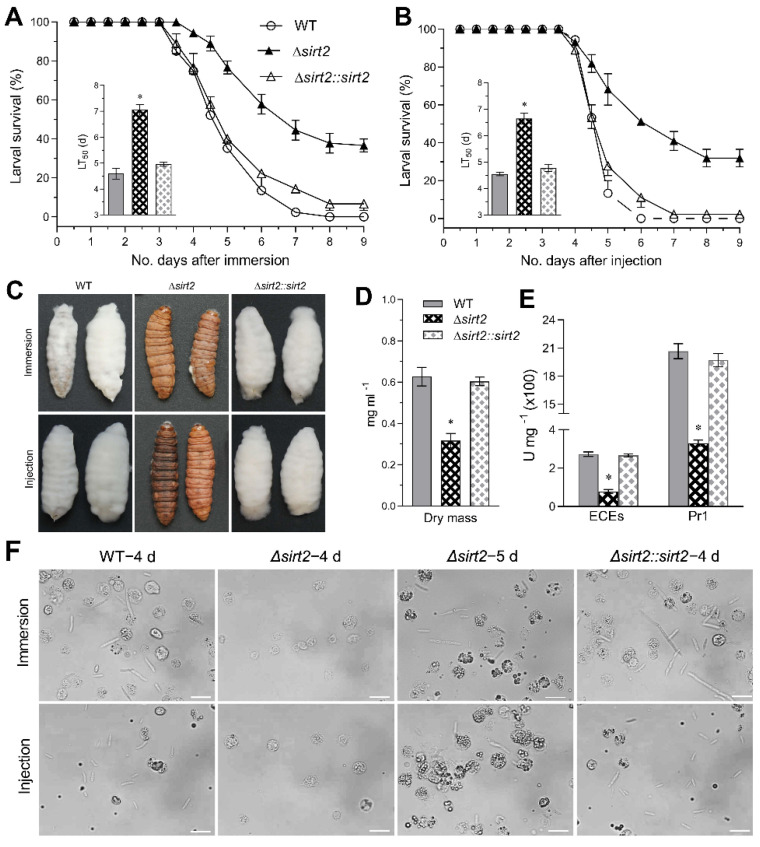
Loss of *BbSirT2* impairs *B. bassiana* virulence. (**A,B**) Mortality curves and calculated LT_50_ values using *G. mellonella* larvae as the target host after topical application (immersion in a 10^7^ conidia mL^–1^ suspension) and intrahemocoel injection (500 conidia/larvae). (**C**) Representative images of fungal growth on cadavers 4 d post-death. (**D**) Quantification of fungal biomass from 3-d CZB-BSA cultures. (**E**) Quantification of activities of total extracellular proteases (ECPs) and Pr1 proteases (3-d CZB-BSA cultures). (**F**) Representative images of *B. bassiana* hyphal bodies (black arrows) isolated from *G. mellonella* larvae hemolymphs 96 and 120 h post-immersion or post-injection. Spherical cells represent host hemocytes (white arrows). Scale bar = 20 μm. The asterisk in each group indicates a significant difference from the unmarked bars (Tukey’s HSD test; * indicates *p* < 0.05). Error bars = ±SD from three replicates.

To assess the contribution of BbSirT2 to *B. bassiana* virulence, larvae of the Greater wax moth *Galleria mellonella* were used as hosts in both intrahemocoel injection (bypassing the cuticle barrier) and topical (natural route of infection) bioassays (Figure 4A,B). In the topical bioassays, the average median lethal time to kill 50% of the target hosts (LT_50_) for the control wild-type strain/complemented strains was ~4.6–5.0 d, while for the Δ*BbSirT2* strain the LT_50_ was ~7.1 d (a 54% increase, i.e., decreased virulence, *p <* 0.001). Using intra-hemocoel injection, the LT_50_ for the wild-type and complemented strains was ~4.5–4.8 d, whereas the LT_50_ for the Δ*BbSirT2* strain was ~6.6 d (a 46% increase, *p <* 0.001).

Visual inspection of cadavers 4 d post-death indicated significantly reduced growth of the infecting fungus on corpses for the Δ*BbSirT2* strain when compared with controls, with minimal mycelial growth seen on Δ*BbSirT2* cadavers (Figure 4C). When grown on CZB media, the fungal mass (total, dry) was ~49% lower (*p <* 0.001) for the Δ*BbSirT2* mutant strain when compared with the wild-type and complemented strains (Figure 4D). After normalization to this reduction in growth, an approximately 70%–85% (*p <* 0.001) decrease in azocasein activity (as a measurement of protease activity) and Pr1 protease activity was noted (Figure 4E). Normal fungal infection of insect hosts leads to the production in vivo (within the insect) of fungal hyphal bodies. Microscopic examination of infected larvae 96 h post intrahemocoel injection or post topical application by the wild-type and complemented strains revealed an abundance of fungal cells in the hemolymph, which were essentially absent in larvae infected by the Δ*BbSirT2* mutant at 4 d post-inoculation but could be detected at 5 d post-infection (Figure 4F).

### 3.5. Role of BbSirT2 in Cell Cycle and Hyphal Septation Regulation

**Figure 5:**
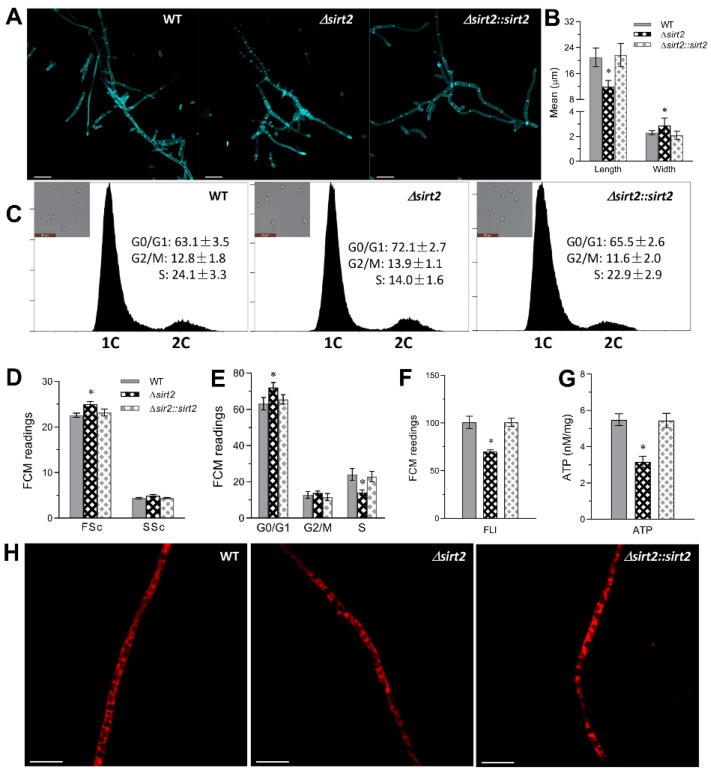
Effect of *BbSirT2* loss on cell cycle progression and hyphal septation. (**A**) Representative microscopic images of *B. bassiana* hyphal cells derived from 72-h SDB cultures and stained with calcofluor white. Scale bar = 20 µm. (**B**) Mean cell width and length analyses of *B. bassiana* calcofluor-white-stained hyphal cells. (**C**,**E**) FACS analysis of the cell cycle phase distribution of *B. bassiana* blastspores isolated from NLB liquid cultures (3 d, at 25 °C). (**D**) FACS analyses examining cell size (FSc) and density (SSc). (**F**) Mito-Tracker Green quantification of mitochondrial content as detailed in the Methods section. (**G**) Quantification of the total ATP content in *B. bassiana* blastspore extracts. (**H**) Representative imags of the mitochondrial distribution in *B. bassiana* hyphae (3-d SDB cultures) visualized via staining with 500 nM Mito-Tracker Red. Scale bar = 10 µm. The asterisk indicates a significant difference from the unmarked bars (Tukey’s HSD test; * indicates *p* < 0.05). Error bars = ±SD from three replicates.

Microscopic examination of fungal hyphae harvested from 72-h SDB cultures and subsequently stained with calcofluor white (a cell wall/chitin-specific dye) revealed an abnormal hyphal septation distribution for the Δ*BbSirT2* strain in comparison with the control strains (Figure 5A). Hyphae derived from Δ*BbSirT2* were significantly shorter (~43%, *p* < 0.01) as well as wider (~33%, *p* < 0.01) than the parental and complemented strains (Figure 5B). To examine effects of BbSirT2 on cell cycle progression, fungal strains were incubated for 3 days at 25 °C in nitrogen-limited broth (NLB) in order to collect unicellular blastospores for analyses. The resultant blastospores were subjected to flow cytometry analyses after propidium iodide (a DNA-specific dye) staining. Blastospores of the Δ*BbSirT2* mutant strain showed a slight but significant increase in size (~11%, *p <* 0.01) and overall cell density (~12%, *p <* 0.01) as compared with the control strains (Figure 5C,D). In addition, Δ*BbSirT2* cells showed slight but significantly longer G_0_/G_1_ phases (~12%, *p <* 0.05), but a more pronounced and shorter S phase (~42%, *p <* 0.01, Figure 5C,E).

Mitochondrial staining (Mito-Tracker Green) coupled with flow cytometric analyses also showed an ~30% decrease (*p <* 0.001) in the total mitochondrial content for Δ*BbSirT2* cells as compared with controls (Figure 5F). To determine any impact the observed change in mitochondrial content might have on ATP production, total ATP levels were measured in cells as detailed in the Methods section. These data show that the total ATP pool was ~42% (*p <* 0.001) lower in Δ*BbSirT2* cells (Figure 5G), and further visualization of hyphal cells stained with Mito-Tracker Red (a mitochondria-specific dye) revealed reduced mitochondrial staining in Δ*BbSirT2* cells (72 h, SDB) as compared with the wild-type and complemented strains (Figure 5H).

### 3.6. Global Gene Expression Changes in the ΔBbSirT2 Mutant

**Figure 6:**
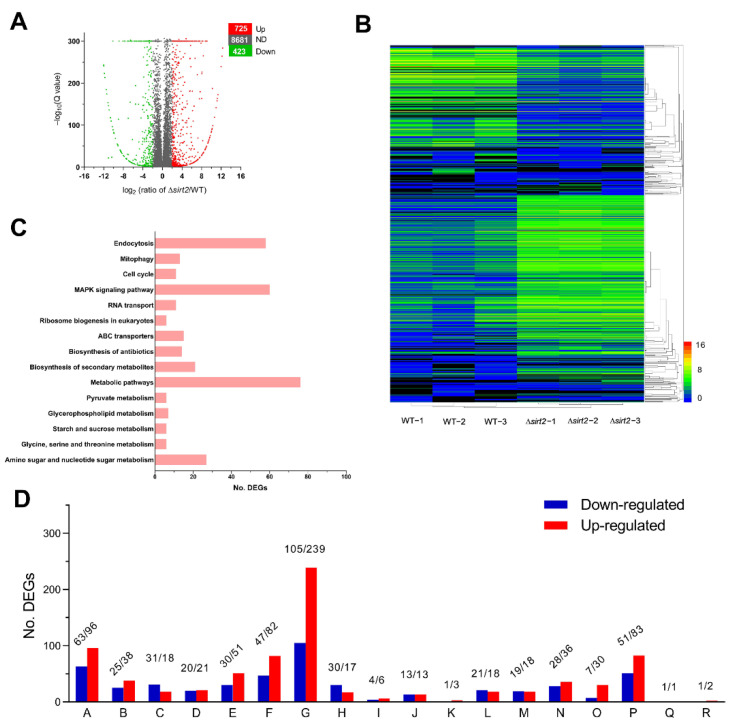
Analyses of the BbSirT2-specific transcriptome. (**A**) Distributions of *p* values and ratios for the genes significantly downregulated, upregulated, and not differentially regulated (ND) in Δ*BbSirT2* versus WT strains grown at 25 °C. (**B**) Cluster analyses of differentially expressed genes (DEGs). (**C**) Total DEG counts enriched in the top 15 KEGG pathways. (**D**) FunCat annotation of 18 functional categories for the set of significantly regulated genes comparing Δ*BbSirT2* to the wild-type parent. A: proteins with a cofactor requirement or involved in binding functions; B: cellular component biogenesis; C: transcription; D: differentiation and cell fate/type; E: protein fate; F: cell defense, rescue, and virulence; G: metabolic processes; H: DNA processing and the cell cycle; I: protein synthesis; J: regulation of protein function and metabolism; K: cell development; L: signal transduction and cell communication; M: cell fate; N: environmental interactions; O: ATP/energy; P: cell transport; Q: systemic environmental interactions; R: viral and plasmid proteins or transposable elements.

Transcriptomes comparing global gene expression between the Δ*BbSirT2* mutant and wild-type strains were constructed and analyzed as detailed in the Methods section, with three independent samples prepared for each strain. The transcriptomic analyses resulted in the identification of 1148 differentially expressed genes (DEGs) in the Δ*BbSirT2* mutant versus WT strains (Tables S3 and S4), including 423 downregulated ((Log_2_ (ratio): −12.02 to −2.00)) and 725 upregulated ((Log_2_ (ratio): 2.00 to 12.68)) genes (Figure 6A,B). The significantly down- and upregulated genes represented 4.1% and 7.0% of the fungal genome [38], respectively. About 43% of the DEGs were classified into 18 different functional classes through FunCat categories classification (Figure 6C). Within the classified DEGs, 70.1% mapped to metabolic processes, 32.4% were involved in a binding or cofactor requirement, 27.3% were involved in cellular transportation, 26.3% mapped to cell rescue, defense, and virulence, 16.5% were involved in protein fate, 13.0% were involved in environmental interactions, 12.8% mapped to cellular component biogenesis, 10.0% were involved in transcription, 9.6% were involved in DNA processing and the cell cycle, 8.4% were involved in cell type differentiation, 7.9% were involved in signal transduction/cellular communication, 7.5% were involved in energy, 7.5% were involved in cell fate, 5.3% were involved in the regulation of protein function and metabolism, and <3% were involved in protein synthesis, systemic development, transposable elements, or systemic interaction with the environment (Figure 6D).

Within the differentially expressed Δ*BbSirT2* dataset, 74 DEGs were involved in DNA events, DNA transcription, and the cell cycle (Table S5), including 27 genes participating in DNA replication, DNA recombination, and DNA damage repair, such as the histone chaperone Rttp106-like protein, the DNA repair and transcription factor Ada, and the nucleotide excision repair protein RAD7. Moreover, 25 DEGs were involved in cytokinesis and cell cycle control (Table S5), including a number of protein kinases in different pathways and several chitin synthases/chitinases related to hyphal septation. In addition, 49 DEGs were involved in RNA synthesis and transcription, such as mRNA capping enzyme, RNA exonuclease, and many transcriptional factors, including the transcriptional repressor TUP1, a metallothionein expression activator, and one fungi-specific transcription factor.

Moreover, 27 DEGs were involved in cellular signal transduction, including protein kinases participating in different pathways (Table S6), including MAPKKK cascades (e.g., a dual-specificity phosphatase and a tousled-like kinase), the NIK-I-kappaB/NF-kappaB cascade (e.g., the BR serine/threonine-protein kinase and the CMGC/SRPK protein kinase), G-protein-mediated signal transduction (e.g., the GTP-binding protein GTR2), and second-messenger-mediated signal transduction (e.g., the calcium/calmodulin-dependent protein kinase and inositol oxygenase). Fifty DEGs were identified that participate in cell differentiation, asexual development (Table S7) (e.g., the FluG protein, the conidiation-specific protein Con-13, and sporulation-associated protein), and cell morphogenesis (e.g., the Bud10 protein and two-cell-surface proteins).

A total of 117 DEGs were identified to be involved in cell defense, rescue, and virulence (Table S8). Some of these genes were related to fungal tolerances to oxidative stress, osmotic stress, and DNA damage stress, including dihydroflavonal-4-reductase protein, flavin-binding monooxygenase-like family protein, chitin synthase 3a, pH regulatory protein, and DNA repair protein. Other DEGs were related to virulence or defense, including a suite of sixteen cytochrome P450s (CYP450), eight major facilitator superfamily (MFS) multidrug transporters, seven ABC multidrug transporters (of the ATP binding cassette type), two polyketide synthases (PKSs), and one LysM domain-containing protein.

Analysis of the DEG set using the pathogen–host interaction (PHI) database revealed 321 DEGs that could be mapped to the database (Table S9). Six genes were associated with the host-lethal phenotype in filamentous fungi. A total of 168 DEGs were involved in reduced virulence or loss of pathogenicity, including 16 cytochrome P450s, 10 ABC multidrug transporters, and 4 MFS multidrug transporters, as also identified above. However, 138 identified DEGs mapped to the “unaffected pathogenicity” subcategory, and 17 DEGs were involved in increased virulence, including 4 cytochrome P450s (CYP450) and 2 chitinases. Interestingly, there were four DEGs associated with avirulence determinant effectors in plant pathogens, including three LysM-domain-containing proteins.

Aside from the results of the KEGG pathway enrichment analysis, 502 DEGs were found to be enriched within 89 KEGG pathways (detailed in Table S10). Within the top 15 KEGG enrichment pathways (Figure 6C), most of the differentially regulated genes were mappped to carbohydrate metabolic pathways, including amino and nucleotide sugar metabolism, pyruvate metabolism, and starch and sucrose metabolism. Many DEGs were associated with metabolism of various amino acids, including serine, glycine, and threonine metabolism, and involved in lipid metabolism (e.g., glycerophospholipid metabolism). In addition, other differentially regulated genes were mainly involved in endocytosis, membrane transport, and signal transduction, including a set of ABC transporters and several protein kinases in the MAPK signaling pathway (Figure 6D).

## 4. Discussion

The acetylation and deacetylation of protein targets affect virtually all cellular processes but have also been shown to be linked to (fungal) virulence [51]. Histone deacetylases, HDACs, and, more generally, KDACs are enzymes that mediate lysine deacetylation and are known to contribute to the regulation of key virulence determinants important to successful infection by various human and animal pathogenic fungi, including *Aspergillus fumigatus, C. albicans*, and *C. neoformans*, as well as different plant pathogenic fungi, including *M. orzyae* and *Fusarium graminearum* [52,53,54,55]. In *B. bassiana*, two class I HDACs, namely the Rpd3 and Hos2 histone deacetylases, and a class III HDAC (Sir2) are known to contribute to cellular homeostasis and virulence [37,56,57]. However, the functional characterization of the remaining class III enzymes remains to be performed, and the extent to which different enzymes contribute to cellular physiology and virulence remains unknown. Here, our results show that, in *B. bassiana*, BbSirT2 plays important roles in asexual development, metabolism, multistress tolerance, and pathogenesis and contributes to the regulation of a large gene set.

Despite the pleiotropic nature of the functionality of BbSirT2 (as revealed by the range of phenotypes observed), specific phenotypic consequences were noted for loss of *BbSirT2* mutants. In applications of entomopathogenic fungi for pest control in the field, conidia represent the typically used active ingredient and the viability of these cells, i.e., their ability to resist environmental stresses, represents a critical parameter in successful pest control applications [34]. As determined by fundamental research on other filamentous fungi, the basic protein products mediating conidiation include the central pathway activators AbaA and BrlA, the upstream FlbA/FlbB/FlbC/FlbD/FlbE and FluG conidiation pathway components, and the downstream regulators/activators VosA and WetA [49,50]. In *B. bassiana*, asexual development and conidiation are severely impaired in mutants of either the *BrlA*, *AbaA,* or *WetA* gene [58]. Our data show that, in the Δ*BbSirT2* mutant, conidiation was decreased by >90% and that any conidia produced appeared to be aberrant in nature. Conidia derived from the Δ*BbSirT2* mutant showed slower germination, reduced hydrophobicity, and reduced UV-B resistance. Transcript levels of *FluG* and *FlbB/D*, whose protein products mediate *BrlA* activation, were significantly reduced along with the expression of the *AbaA*, *BrlA,* and *VosA* genes. This was consistent with the reduced expression of the *FluG* and *BrlA* genes observed in the global transcriptomic analyses. Moreover, the expression of the hydrophobic *Hyd2,* which mediates conidial cell surface hydrophobicity and is involved in conidial adherence to the host cuticle [59], was also downregulated in the Δ*BbSirT2* mutant strain. The transcriptional repression of these genes associated with conidiation and conidial development provides the likely reason for the atrophied conidial production in the Δ*BbSirT2* mutant strain.

Regarding filamentous growth, hyphal septation and ordered cell cycle progression are critical for spore development and vegetative growth. Normal hyphal formation also impacts fungal pathogen infection, since these structures often mediate or differentiate into infectious structures. We found that loss of *BbSirT2* resulted in blocked hyphal septation and distorted hyphal cell and blastospore morphologies coupled with abnormal (delayed) cell cycle progression, i.e., a shorter S phase but a significantly longer G_0_/G_1_ phase. The aberrant hyphal septation and altered cell cycle phenotypes observed were consistent with the observed repression of the expression of several septation-related genes [60], including *ChsB* and *CsmA* in the *BbSirT2*-specific transcriptome. Moreover, several genes related to cell cycle control and G–S transition were found to be significantly downregulated in the Δ*BbSirT2* mutant as compared with the wild-type parent, including two cyclin-dependent kinases (Cdks) involved in cell cycle progression.

Specific sirtuin HDACs have been shown to contribute to normal hyphal growth and virulence in *C. albicans* [29,61,62] and *C. neoformans* [30,63]. In *M. orzyae*, the sirtuin MoSir2 contributes to biotrophic growth and participates in the inhibition of host plant defense responses, including the formation of reactive oxygen species (ROS) [64]. In our study, virulence was severely impaired in the Δ*BbSirT2* mutant in insect bioassays involving both the cuticle-bypassing (intrahemocoel injection) infection route and the “normal” host topical insect cuticle infection route, suggesting that BbSirT2 contributes to both cuticle penetration and subsequent immune evasion processes. With respect to the former, the Δ*BbSirT2* strain was shown to have reduced secretion of extracellular cuticle-degrading protease enzymes involved in cuticular penetration [65]. With respect to the former, we observed a decrease in the rate of hyphal body formation resulting from the dimorphic transition of the penetrating hyphae into free-floating (within the hemocoel) fungal cells [66,67]. Consistent with the decreased virulence phenotype, genes involved in cuticular penetration and/or the fungal dimorphic transition of *B. bassiana* were significantly downregulated in the Δ*BbSirT2* strain. Apart from the downregulation of *Hyd2*, three LysM-domain-containing proteins, two polyketide synthases (PKSs) involved in secondary metabolism and fungal pathogenicity, and the *AbaA*, *BrlA*, and *Bud4* genes implicated in virulence [68,69,70] were also downregulated in the Δ*BbSirT2* mutant as compared with the wild-type parent. Moreover, a number of genes involved in cell defense and rescue that likely impact fungal virulence by mediating cellular responses to host immunity in *B. bassiana* were significantly downregulated in the Δ*BbSirT2* mutant [36]. This included the repression of the expression of two P-type Na^+^/K^+^ ATPases, *Ena1c*, and *Ena2b*, which contribute to the maintenance of cellular homeostasis and fungal tolerances to various abiotic stresses, e.g., osmotic stress and UV irradiation [71]. Moreover, the transcription of four ABC multidrug transporters and one MFS multidrug transporter required for conidial tolerance to antifungal drugs [72] was differentially downregulated as well. This transcriptional repression of genes associated with fungal infection and resistance to multiple stresses in the host hamocoel indicates important roles for *BbSirT2* in sustaining fungal virulence and the biological pest control potential of *B. bassiana*.

Loss of *BbSirT2* reduced the ability of the fungus to efficiently utilize a range of different carbon and nitrogen sources. In the BbSirT2-specific transcriptome, 344 genes involved in metabolism were down- or upregulated, accounting for 30% of the differentially regulated genes in Δ*BbSirT2*. Among these genes, 105 genes were downregulated, most of which were enriched in the processes of nitrogen and carbon metabolism, lipid metabolism, and amino acid metabolism. The downregulation of these genes concurred with the severe growth defects in Δ*BbSirT2* on all of the tested media containing different kinds of carbon (sugar/polyol) or nitrogen sources. These transcriptomic data indicate that BbSirT2 orchestrates the processes of nitrogen and carbon metabolism by transcriptional regulation of related genes contributing to the uptake of nutrients from the envionment and regulating metabolic processes in *B. bassiana*. Our results reinforce the conserved role that sirtuins play in mediating the regulation of nitrogen and carbon source utilization, including in mammals, plants, and bacteria [1,2,3,73]. Our data indicate that loss of *BbSirT2* reduces ATP levels via impacting (reducing) cellular mitochondrial content. Consistent with this phenotype, 37 DEGs were identified to be related to energy generation and ATP synthesis, e.g., ATP synthase subunit beta, ATP synthases, and the ADP and ATP carrier protein. Overall, BbSirT2 appears to help mediate the regulation of mitochondrial biosynthesis and energy homeostasis in *B. bassiana*. Our data show that BbSirT2 performs imporant functions in regulating the expression of a wide range of genes, including those essential for normal cell cycle control and progression, asexual development, tolerances to various stress conditions, and the virulence of *B. bassiana*.

## 5. Conclusions

Within the context of the *B. bassiana*–insect host–pathogen system, our data show that the BbSirT2 class III histone deacetylase pleiotropically affects a wide range of gene targets, impacting conidiogenesis, development of and progression through the cell cycle, metabolic use of nitrogen and carbon sources, stress responses, and pathogenesis. Over 381 putative targets belonging to uncharacterized proteins were identified, suggesting a set of targets that remain uncharacterized. Despite the array of targets, and the expected pleiotropic effects, discrete alterations in gene expression, including in the expression of LysM effectors and other pathogenicity-related genes, were found. These data expand our understanding of sirtuins’ functions in the maintenance of cell homeostasis and differentiation and their contributions to fungal growth, stress responses, and virulence.

## Data Availability

All transcriptomics data have been deposited in the Sequence Read Archive (SRA) on the NCBI website (https://www.ncbi.nlm.nih.gov/, accessed on 22 December 2020) with the dataset identifier PRJNA687276.

## Supplementary Materials

The following supporting information can be downloaded at https://www.mdpi.com/article/10.3390/jof8030236/s1. Figure S1. *B. bassiana* SirT2 phylogenetic analysis with homologs identified in other representative organisms. Figure S2. Construction and verification of *B. bassiana SirT2* mutants. Figure S3. Stress response phenotype of Δ*BbSirT2* mutant and control strains. Table S1. Lists of primers used for the *BbSirT2* mutant strain’s construction. Table S2. List of primers used for qPCR transcriptional profiling. Table S3. Lists of 1148 differentially expressed genes (DEGs) in the Δ*Bb**SirT2* mutant compared with the wild-type parent. Table S4. The FRKMs in the DEG heatmap of the Δ*BbSirT2* mutant and wild-type strains. Table S5. Lists of 74 BbSirT2-regulated genes involved in DNA events, the cell cycle, and transcription in *B. bassiana*. Table S6. Lists of 27 DEGs involved in signal transduction and cell communication in the Δ*BbSirT2* mutant. Table S7. Lists of 50 DEGs involved in morphogenesis and cell type differentiation in the Δ*BbSirT2* mutant. Table S8. Lists of 117 DEGs involved in cell defense, rescue, and virulence in the Δ*BbSirT2* mutant. Table S9. Lists of 321 DEGs identified in the Δ*BbSirT2* mutant using the pathogen–host interaction (PHI) database analyses. Table S10. Lists of 502 DEGs mapped to 89 KEGG pathways in the Δ*BbSirT2* mutant. Table S11. Lists of 37 DEGs involved in energy homeostasis in the Δ*BbSirT2* mutant.
