# Supplementary material for: Contributions of a Histone Deacetylase (SirT2/Hst2) to Beauveria bassiana Growth, Development, and Virulence
Source: J Fungi (Basel). 2022 Feb 27;8(3):236. doi: 10.3390/jof8030236 (PMC8950411; doi:10.3390/jof8030236)
Supplement: Supplementary file 1 [file jof-08-00236-s001.zip › Supplementary Material-Minor.pdf]

Supporting Information:

Contributions of a histone deacetylase (*SirT2/Hst2*) to *Beauveria bassiana* growth, development, and virulence

Qing Cai[1, 2*], Li Tian[3], Jia-Tao Xie[1], Dao-Hong Jiang[1] and Nemat O. Keyhani[2*]

[1] State Key Laboratory of Agricultural Microbiology, College of Plant Science and Technology, Huazhong Agricultural University, Wuhan, Hubei, 430070, China.

[2] Department of Microbiology and Cell Science, University of Florida, Bldg. 981, Museum Rd., Gainesville, FL, 32611, USA.

[3] Shandong Provincial Key Laboratory of Microbial Engineering, Department of Bioengineering, Qilu University of Technology, Jinan, Shandong, 250353, China

* Correspondence: **author:** Qing Cai, E-mail: caiqing@mail.hzau.edu.cn; Nemat, O. Keyhani, E-mail: keyhani@ufl.edu.

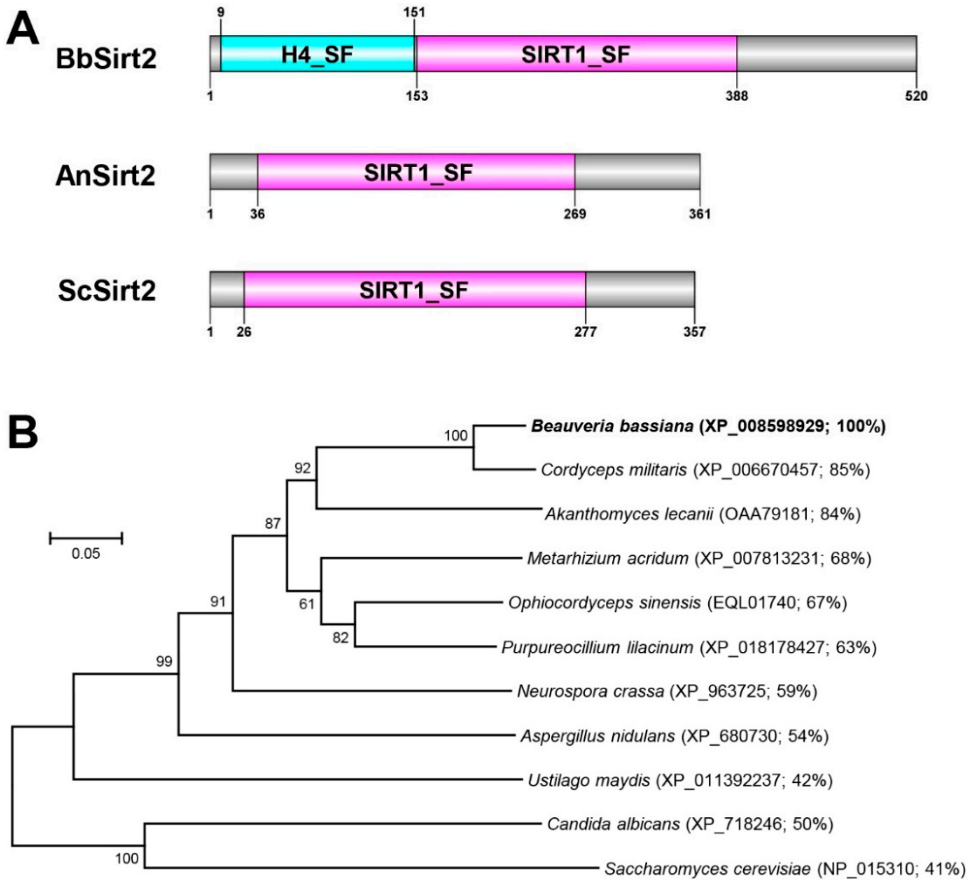

Figure S1. *B. bassiana* SirT2 phylogenetic analysis with homologs identified in other representative organisms. (**A**) Conserved domain of Sirt2 homologs found in *Beauveria bassiana* (*Bb*), *Aspergillus nidulans* (*An*), and *Saccharomyces cerevisiae* (*Sc*) predicted at https://blast.ncbi.nlm.nih.gov/Blast.cgi. (**B**) Phylogenetic analyses of *B. bassiana* Sirt2 with the homologs found in other representative fungi. A neighbor-joining method in MEGA7 software at http://www.megasoftware.net was used in the phylogenetic analysis. Poisson model was performed with 1000 bootstrap replications in uniform rates. Each fungal name is followed by the NCBI accession code of each protein and its sequence identity (%) to *B. bassiana* Sirt2 in parentheses. Scale bar: branch length proportional to genetic distance assessed with the neighbor-joining method.

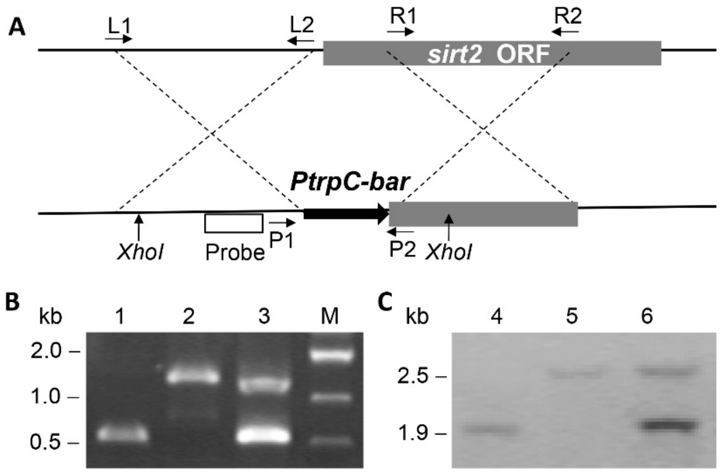

Figure S2. Construction and verification of *B. bassiana* SirT2 mutants. (**A**) Schematic diagram for the strategy of *Sirt2*deletion. (**B, C**) The *Sirt2*mutants identified via PCR (lanes 1–3) and Southern blotting (lanes 4–6) analyses with paired primers and amplified probe (Table S1). Lanes 1 and 4: wild-type. Lanes 2 and 5: Δ*Sirt2* mutant. Lanes 3 and 6: Δ*Sirt2::*

Sirt2 mutant. Genomic DNAs were digested with *Xho*I/*Xho*I at the marked sites for the Southern blotting of *Sirt2*.

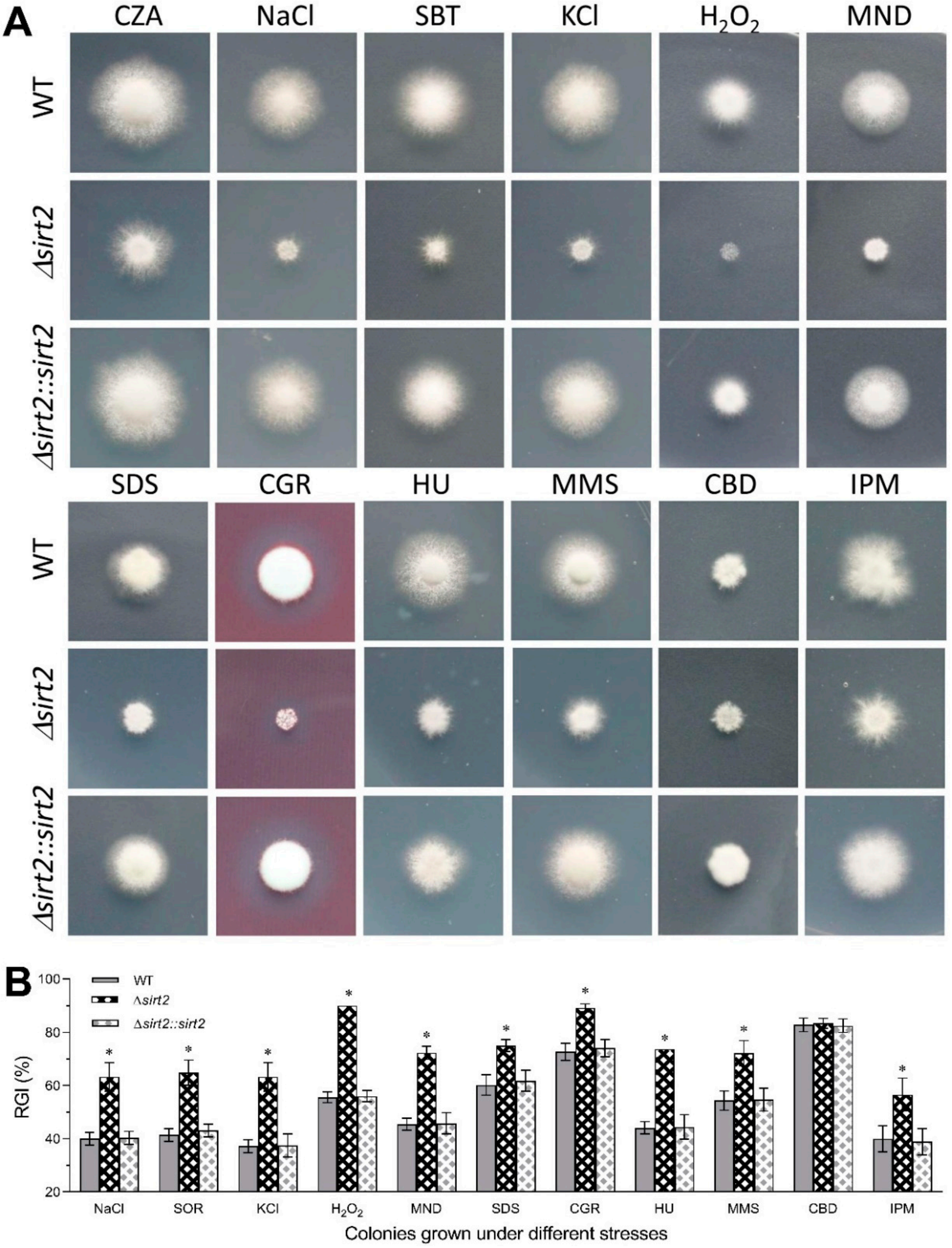

Figure S3. Stress response phenotype of *ΔBbSirT2* mutant and control strains. (**A, B**) Images and RGI (relative growth inhibition) of fungal colonies grown at 25°C for 7 d on CZA supplemented with either, (i) CZA unamended, control, (ii) H_2O_2 (2 mM) or menadione (0.02 mM), oxidative stress (ii) 0.4 M NaCl, 0.4 M KCl or 0.8 M sorbitol, osmotic stress; (iii) H_2O_2 (2 mM) or menadione (0.02 mM), oxidative stress; (iv) carbendazim (CBD) (10 $\mu g\ ml^{-1}$) or iprodione metabolite (IPM, 10 $\mu g\ ml^{-1}$), drug resistance analysis; (v) hydroxyurea (HU) (10mM) or methyl methanesulfonate (MMS) (0.05%), DNA damage stress, (vi) SDS (100 $\mu g\ ml^{-1}$) or Congo red (10 $\mu g\ ml^{-1}$), cell wall perturbing stress. The asteristed bar in each three-bar group differ significantly from those unmarked (Tukey's HSD, $p < 0.05$). Error bars: SD.

Table S1. Lists of primers used for the *BbSirT2* mutant strains construction.

Primers	Paired sequences (5'-3')*	Purpose
Sirt2up-F/R	AAAAACCCGGGCACTGTCTTTTGCTTCTCCGTC/ AAAAAGGATCCGACAAAAGTAAGCGTGCGTGG	Cloning *Sirt2* 5′-end (1579 bp) for *Sirt2* deletion
Sirt2dn-F/R	AAAAATCTAGACTGACCTACAAACAGCTACCGT/ AAAAAACTAGTTCGTCAGGGAACTCTTTTTG	Cloning *Sirt2* 3′-end (1576 bp) for *Sirt2* deletion
Sirt2fl-F/R	ggggACCACTTTGTACAAGAAAGCTGGGTNCATCAC-GGTCCTCATT-GTC/ggggACAAGTTTGTACAAAAAAGCAGGCTNGTCCATCGGTAGCGTTATT	Cloning full-length *Sirt2* (5102 bp) for *Sirt2* complementation
cSirt2-F/R	CCCGGGACTAGTGATATCATGTCGGACCAC-GAATTCGG/CTTGCTCACCATGAATTCAAGGGCCGACTTGGGAAAAC	Cloning *Sirt2* cDNA (1560 bp)
pSirt2-F/R	ACCTATTACTCGCCGTCGCTC / GCAGCATTCTATGAG-CAGGTC	PCR detecting *Sirt2*
sbSirt2-F/R	AGCTCGACTCGGTCATTT / TGCTTGTGCCTTCTTATTC	Southern probe of *Sirt2* (418 bp)
qSirt2-F/R	AAGACTGGGCTGTATAAC / GTAGAATGGCTCAGGATT	qPCR detecting *Sirt2*
q18S-F/R	TGGTTTCTAGGACCGCCGTAA / CCTTGG-CAAATGCTTTCGC	qPCR detecting 18S RNA

* Underlined regions denote the restriction enzyme sites for the deletion of *Sirt2* (*Xma*I/*BamH*I and *Xba*I/*Spe*I) and the cloning of *Sirt2* cDNA (*Eco*RV/*Eco*RI) or the fragments of gateway exchange for *Sirt2* complementation.

Table S2. Primer list used for qPCR transcriptional profiling.

Tag loci*	Gene	Annotation	Paired primers used in qRT-PCR
BBA_04942	*FluG*	developmental protein	CCTCCCTAGTTTGGTCGCTTTCTC / CGCTGTCGGTAATCTGCTCCTC
BBA_02968	*FlbA*	developmental regulator	CCAATCCACTCGCCGCTCTC / CGGAGGAAA-GAGAATCGGTAGAGG
BBA_06988	*FlbB*	bZIP transcription factor	GCACTGACACGCCGACAAGAGC / CCGCCGCCGAAGCCTGTTG
BBA_03181	*FlbC*	C_2H_2 conidiation transcription factor	TCCATCTCCAACTTGCTGGGTCTC / GGCGGCG-TAGGCGGAAGG
BBA_07259	*FlbD*	MYB conidiophore development protein	CGGCAAGCGATGGGCAGAGATTG / ACGAG-CAAGGTGACGGTAGAGGTG
BBA_01716	*FlbE*	conidiophore development protein	CAGACGATGAGACAGAGA/ GGGCTTTA-TATGCGAGTAG
BBA_07544	*BrlA*	C_2H_2 conidiation transcription factor	GACCAGTTCAACAGACAAG / CAG-TAATCTTCGTGCTTCTC
BBA_00300	*AbaA*	Conidiation factor	GCAAGTCTCCAGCCATAT / CTCCTCTTCGTCATAGTAGTC
BBA_06126	*WetA*	Conidial maturation factor	CGCAGACGAATTTGACTT/ GCTGGTGGTT-GAATACAT
BBA_01023	*VosA*	Velvet protein	GGACAGACGAGTGATTGA / GGCATATACGAC-GCATCT

* Gene accession codes in the genome database of *B. bassiana* under the NCBI accession NZ_ADAH00000000.
